# Cutinase-Catalyzed Polyester-Polyurethane Degradation: Elucidation of the Hydrolysis Mechanism

**DOI:** 10.3390/polym14030411

**Published:** 2022-01-20

**Authors:** Federico Di Bisceglie, Felice Quartinello, Robert Vielnascher, Georg M. Guebitz, Alessandro Pellis

**Affiliations:** 1 Department of Agrobiotechnology, University of Natural Resources and Life Sciences Vienna, 3430 Tulln an der Donau, Austria; federico.dibisceglie@gmail.com (F.D.B.); robert.vielnascher@boku.ac.at (R.V.); guebitz@boku.ac.at (G.M.G.); 2 Austrian Centre of Industrial Biotechnology, 3430 Tulln an der Donau, Austria; 3 Dipartimento di Chimica e Chimica Industriale, Universitá degli Studi di Genova, Via Dodecaneso 31, 16146 Genova, Italy

**Keywords:** plastic degradation, polyurethanes, enzyme catalysis

## Abstract

Polyurethanes (PU) are one of the most-used classes of synthetic polymers in Europe, having a considerable impact on the plastic waste management in the European Union. Therefore, they represent a major challenge for the recycling industry, which requires environmentally friendly strategies to be able to re-utilize their monomers without applying hazardous and polluting substances in the process. In this work, enzymatic hydrolysis of a polyurethane-polyester (PU-PE) copolymer using *Humicola insolens* cutinase (HiC) has been investigated in order to achieve decomposition at milder conditions and avoiding harsh chemicals. PU-PE films have been incubated with the enzyme at 50 °C for 168 h, and hydrolysis has been followed throughout the incubation. HiC effectively hydrolysed the polymer, reducing the number average molecular weight (M_n_) and the weight average molecular weight (M_w_) by 84% and 42%, respectively, as shown by gel permeation chromatography (GPC), while scanning electron microscopy showed cracks at the surface of the PU-PE films as a result of enzymatic surface erosion. Furthermore, Fourier Transform Infrared (FTIR) analysis showed a reduction in the peaks at 1725 cm^−1^, 1164 cm^−1^ and 1139 cm^−1^, indicating that the enzyme preferentially hydrolysed ester bonds, as also supported by the nuclear magnetic resonance spectroscopy (NMR) results. Liquid chromatography time-of-flight/mass spectrometry (LC-MS-Tof) analysis revealed the presence in the incubation supernatant of all of the monomeric constituents of the polymer, thus suggesting that the enzyme was able to hydrolyse both the ester and the urethane bonds of the polymer.

## 1. Introduction

Polyurethanes (PU) are a class of polymers obtained from the polycondensation reaction of a di-isocyanate and a polyol in the presence of an organic or organometallic catalyst or through ultraviolet light activation [1,2]. Polyurethanes were originally synthesized by Otto Bayer in 1937 using di-functional hexane di-isocyanate and 1,4-butanediol as monomers, obtaining stiff fibres that could be used for brush bristles [3]. Thanks to their wide range of properties, PU have many different potential applications, such as fibres, adhesives and shape-memory materials [4,5]. Originally, these polymers contained only a characteristic intra-molecular urethane bond (carbonate ester bond -NHCOO-) [6], but more recently they were modified to also include ester or ether bonds in their structure, therefore expanding their properties and fields of application [7]. The incorporation of short-chain polyol with a high level of crosslinking results in rigid and tough polymers, while soft and stretchy PU can be obtained using flexible and long chain polyols having a low degree of crosslinking [8,9]. A combination of hard and soft segments can also be obtained within the same macromolecule, thanks to the exploitation of the different cross-linking abilities of the various monomers. This happens, for example, in thermoplastic polyurethanes, a versatile class of polymers that finds application as coatings, drug-release controlling systems and polymer blends [10]. The main form in which PU are used are foams. Generally, these materials are obtained when water is present in the reaction mixture, since the carbamic acid formed by the addition of water to the isocyanate molecules is unstable and releases gaseous CO_2_, which forms bubbles that lead to the formation of pores in the material’s structure [11]. Polyurethanes foams are commonly used in the automotive, packaging and construction industries [12].

From the waste management point of view, due to their peculiar physical properties, PU foams are included in a separate group from the PU used as CASE (Coatings, Adhesives, Sealants, Elastomers) [13]. Substantially, PU foams act differently from fibres and other polymer formulations, especially in landfill scenarios, since they have larger volumes due to their low density and the high amount of air inside them that might cause the development of deep-seated fires. Currently, landfilling is the main strategy for the disposal of end-of-life PU, accounting for almost half of the whole European PU production [14]. PUs themselves represent almost 8% of the produced plastics in Europe, being the sixth most used plastic [15] and accounting for almost 25% of plastic waste management in the European Union [14].

With plastic recycling receiving increasing attention due to the enforcement of new, more stringent environmental legislations, the reuse of PU has also become an important point to tackle. One way to recycle PU polymers is mechanical shredding of scrap polymers into fine powders to be used as fillers in newly manufactured materials. This technique, however, cannot be applied to foams due to their high level of crosslinking [16] and post-consumer waste products cannot be the stock for mechanical recycling due to the presence of contaminants or the addition of other materials [17]. Another way to recycle PU waste is through chemical processes, which allow us to reverse the polymerization reactions, recovering some building blocks that can be reused for polymer synthesis or in other unrelated operations, such as the production of syngas [18]. Among these processes, there are hydrolysis, phosphorolysis and glycolysis. Hydrolysis is the reaction between PU waste and water in the presence of ammonia or sodium hydroxide as catalysts, resulting in polyols, amine intermediates and CO_2_ [19], while phosphorolysis is a reaction in which esters of phosphonic or phosphoric acids react with the urethane groups, yielding phosphorus-containing oligourethanes that can be used in the production of new polymers with flame-retardant properties [20]. Last but not least, glycolysis is the most used chemical recycling method for rigid and flexible PUs and comprises a transesterification reaction in which a hydroxyl group from a glycol replaces the polyol in the urethane bond, yielding reduced molecular weight oligomers [21].

To avoid the use of chemical catalysts and to achieve decomposition at milder conditions, therefore reducing operational costs and avoiding harsh and polluting chemicals, enzymatic depolymerisation of PU has been the object of preliminary research. Moreover, enzymes, due to their high specificity, would allow stepwise recovery of buildings blocks even from blended/multi-layer materials. The main limitation to this approach is the fact that PUs do not occur in nature, and so the number of known enzymes able to hydrolyse this class of materials is still very limited [22,23]. Despite this drawback, a number of enzymes, belonging to different classes of hydrolases, has been reported to hydrolyse PU. Some examples include an esterase from *Comamonas acidovorans* TB-35 [24] and the polyester hydrolysing enzymes TfCut2, Tcur0390 and Tcur1278 isolated from *Thermobifida fusca* KW3 and *Thermomonospora curvata* DSM43183, respectively, as well as the LC cutinase [7]. A combination of an esterase and an amidase has also been reported to degrade thermoplastic polyurethanes by both ester bond hydrolysis and urethane bond cleavage [23]. The ability of esterases, lipases and amidases to hydrolyse carbamate bonds has also been shown by Zadło-Dobrowolska et al., who inserted a carbamate bond into their probes, designed for screening the activity of hydrolytic enzymes [25]. Among other classes of hydrolases, the protease papain was found to be effective in degrading PU [26], and so was porcine pancreatic elastase [27]. A novel approach involved the fusion of a hydrophobic binding module from a poly(hydroxyalkanoate) depolymerase from *Alcaligenes faecalis* to a polyamidase from *Nocardia farcinica*. The activity of the fusion enzyme increased, suggesting that enzyme adsorption seems to play a major role in enhancing PU hydrolysis [22].

Cutinases (EC 3.1.1.74) belong to the α/β hydrolase superfamily. They possess a classical Ser–His–Asp catalytic triad. This class of enzymes is known to be able to hydrolyse high-molecular-weight synthetic polyesters such as poly(butylene succinate) (PBS), poly(1,4-butylene 2,5-furandicarboxylate) (PBF), poly(ethylene 2,5-furanoate) (PEF) and poly(ethylene terephthalate) (PET) [28,29,30,31,32,33]. Moreover, cutinases can catalyse esterification and transesterification reactions on both small substrates and polymers [34,35], therefore showing a wide range of applications in the environmental, chemical, detergent and textile industries [36]. Some industrial examples involve the biodegradation of polymer waste [37] and the surface functionalization and degradation of natural and synthetic fibres, including cotton [38] and polyamides [39]. Recent studies have been dedicated to the comparison between cutinases and lipases, which are already extensively used in industrial applications, to evaluate the feasibility of a practical application of these polymers in commercial processes [40]. However, there is only very little information available about polyurethane-polyester (PU-PE) hydrolysis and related reaction pathways. Therefore, in the present work, the hydrolysis of PU-PE copolymers has been investigated utilising the cutinase from *Humicola insolens* (HiC). A detailed characterization of the hydrolysis mechanism and the analysis of the released products was carried out using gel permeation chromatography (GPC) and LC-TOF/MS.

## 2. Materials and Methods

### 2.1. Chemicals, Substrates and Enzymes

Buffer components and model substrates such as para-nitrophenyl-butyrate (pNPB), para-nitrophenyl-*N*-benzylcarbamate (pNPC), butyl-*N*(para-nitrophenyl)carbamate (pNPBC) and para-nitrobutyranilide (pNPA) were purchased from Sigma-Aldrich (Vienna, Austria). All other chemicals and reagents used in this work were of analytical grade and used without further purification if not otherwise specified. Humicola insolens cutinase (HiC) was purchased from STREM chemicals (BISCHHEIM, France, product code 06-3135, Novozym 51032, CAS Number: 9001-62-1). Commercial polyurethane pellets (PU1080) were kindly provided by Bayer (Leverkusen, Germany).

### 2.2. Enzyme Characterization

The protein concentration was determined with the Biorad protein assay (Bio-Rad laboratories GmbH, Vienna, Austria) as previously described [32] (Figure S6).

Hydrolytic activity was primarily tested on different model substrates, specifically para-nitrophenyl-butyrate (pNPB) (Figure S1A), para-nitrophenyl-*N*-benzylcarbamate (pNPC) (Figure S1B), butyl-*N*(para-nitrophenyl)carbamate (pNPBC) (Figure S1C), and para-nitrobutyranilide (pNPA) (Figure S1D).

The increase in the absorbance at 405 nm (for *p*-NPB and p-NPC) and 385 nm (for pNPBC and pNPA) was measured using a Tecan INFINITE M200 plate reader (Männedorf, Switzerland). A blank was measured using 20 μL of buffer instead of sample. The increase in the absorbance (at 25 °C) indicated an increase in p-nitrophenol and p-nitroaniline (ε405 nm = 7.8 mmol^−1^ cm^−1^, ε385 nm = 9.25 mmol^−1^ cm^−1^). The activity was calculated in units, where 1 unit had been defined as being the amount of enzyme required to hydrolyse 1 μmol of substrate per minute under the given assay condition. The stock solution concentration of HiC was determined as 11.5 mg mL^−1^ (±0.11 mg mL^−1^) and further diluted in buffer till the linear range of enzymatic activity was observed; for p-NPB activity assay, for example, the enzyme was diluted in buffer with ratio 1:5000.

### 2.3. PU-PE Films Production

To obtain films suitable for hydrolysis testing, PU-PE pellets were solubilised in 1 mL of THF at a concentration of 25 mg mL^−1^. The polymer solution was casted in 20 mL vials and THF was then left to evaporate overnight, resulting in 25 mg of thin PU-PE films.

### 2.4. PU-PE Hydrolysis

A total of 25 mg of PU-PE film were incubated in triplicate in 5 mL of 1 M potassium-phosphate buffer at pH 8.0-containing HiC at a concentration of 0.5 mg mL^−1^. The solution was incubated at 50 °C and 150 rpm. Reactions were stopped at various time points (1, 24, 48, 72, 120 and 168 h) removing the enzyme (or blank) solution and freeze-drying the vial with the leftover PU-PE film before carrying out the various characterizations.

### 2.5. Liquid Chromatography Time-of-Flight/Mass Spectrometry (LC-TOF/MS)

A total of 2 mL of each sample was centrifuged at 12,700 rpm and 4 °C for 15 min and the supernatant was filtered in PTFE filters with a cut-off of 0.2 μm. Liquid chromatography time-of-flight/mass spectrometry (LC-TOF/MS) in positive ionization mode was used to qualitatively identify the released soluble oligomers. The analytes were separated using an HPLC (1260 series, Agilent Technologies, Palo Alto, CA, USA) equipped with a reversed-phase C18 rapid resolution column (Zorbax Eclipse XDB, Agilent Technologies, Palo Alto, CA, USA) of 50 mm by 2.1 mm and 1.8 µm particle diameter at a total runtime of 15 min. Column temperature was 40 °C. Mobile phase A consisted of 20 mM NH_4_COOH in ultrapure water and mobile phase B was MS-grade acetonitrile.

The flow rate was 0.5 mL min^−1^ and the injection volume was 20 µL. This HPLC system was connected to a time-of-flight mass spectrometer (G6230B, Agilent Technologies, Palo Alto, CA, USA) equipped with a dual electrospray ionizer (ESI) under the following operating conditions: capillary 3500 V, nebulizer 40 psig, drying gas 8 L min^−1^, gas temperature 300 °C, fragmentor 125 V, skimmer 65 V, OCT 1 RF Vpp 750 V. The mass axis was calibrated using the mixture provided by Agilent Technologies over the mass range of 50–3200 *m/z*. In addition to the second orthogonal nebulizer supplied with a reference mass solution was used as a continuous calibration using the following reference masses: 121.05087 and 922.00979 *m/z*. Spectra were acquired with the Agilent Technologies software MassHunter (Version 10.1) over the 50–3000 *m/z* range at a scan rate of two spectra per second (Figures S2–S5).

### 2.6. Gel Permeation Chromatography (GPC)

The film samples for each timepoint were frozen at −20 °C and lyophilised. Each sample was then dissolved in THF at a concentration of 2 mg mL^−1^ and filtered through a cotton filter prior to passing into a HPLC vial in order to remove the insoluble salts from the aqueous media used to carry out the reaction. Gel permeation chromatography was carried out at 30 °C on an Agilent Technologies HPLC System (1260 series, Agilent Technologies, Palo Alto, CA, USA) connected to a 6.0 mm ID  ×  40 mm L HHR-H, 5 μm Guard column and a 7.8 mm ID  ×  300 mm L GMHHR-N, 5 μm TSK gel liquid chromatography column (Tosoh Bioscience, Tessenderlo, Belgium) using 1 mL min^−1^ THF as the mobile phase. An Agilent Technologies G1362A refractive index detector was employed for detection. The molecular weights of the polymers were calculated using linear polystyrene calibration standards in the 400–2,000,000 Da M_p_ range (Sigma-Aldrich, St. Louis, MO, USA). Data processing was carried out using the Agilent GPC/SEC software (Version 1.2, Figures S7–S10).

### 2.7. FT-IR Analysis

The film samples were washed with 5 mgmL^−1^ Triton X100, 100 mM Na_2_CO_3_ and ultrapure water sequentially for 30 min for each solution to remove salt residues and other impurities from their surface. The samples were then dried at 60 °C overnight and the spectra were recorded on a PerkinElmer Spectrum 100 spectrometer. Spectra were collected at a resolution of 4 cm^−1^ for 40 scans and normalized at 1413 cm^−1^ before data processing.

### 2.8. Scanning Electron Microscopy (SEM)

PU-PE film morphology was qualitatively assessed through Scanning Electron Microscopy (SEM). Control PU-PE (without any enzymatic treatment) and enzymatically hydrolysed films (after 1, 72, and 168 h) were surface characterized. The fracture surface was sputter coated with a 10 nm layer of platinum to provide sufficient electrical conductivity. All SEM images were acquired collecting secondary electrons on a Hitachi 3030 TM (Metrohm INULA GmbH, Austria) using 5 eV.

### 2.9. ^1^H-NMR Analysis

After freezing and lyophilization, the film samples for each timepoint were dissolved in THF-d_8_. The ^1^H-NMR spectra were acquired using a Bruker Avance II 400 spectrometer (Vienna, Austria) (resonance frequency 400.13 MHz for ^1^H) equipped with a 5 mm observe broadband probe head (BBFO) (Vienna, Austria) with z-gradients.

## 3. Results and Discussion

**Figure 1:**
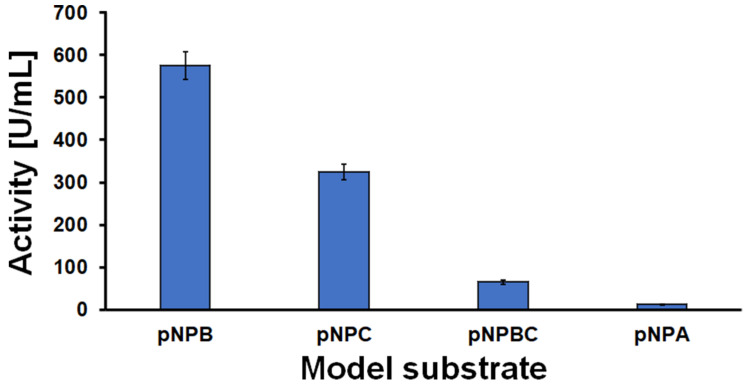
Activity of *Humicola insolens* cutinase on colorogenic model substrates containing ester, urethane, and amide bonds. The activity was calculated in units, where 1 unit had been defined as being the amount of enzyme required to hydrolyse 1 μmol of substrate per minute under the given assay condition.

The hydrolysis activity of *Humicola insolens* cutinase (HiC) towards different bonds such as ester, urethane and amide bonds was evaluated using four model substrates (Figure 1). It can be observed that the activity measured with the substrates presenting an ester bond (pNPB) was higher than the one obtained using substrates containing a urethane or amide bond (pNPC, pNPBC and pNPA).

*Humicola insolens* cutinase demonstrated to be a versatile biocatalyst; as previously shown by Quartinello et al., HiC was used for a nylon surface functionalization application [41].

Urethane bonds represent a hybrid system between ester and amide bonds. Therefore, the different model substrates assays were performed to better understand the affinity of the biocatalysts towards those chemical bonds. Looking in detail, the chemical structure of the model compounds, the different orientation/position of the p-nitrophenol, and subsequentially its release leads to more information about the cleavage site of HiC.

HiC was used to hydrolyse PU-PE films at a concentration of 0.5 mg mL^−1^ in 1 M K_2_HPO_4_/KH_2_PO_4_ buffer at pH 8. The hydrolysis was carried out at 50 °C and for a maximum of 7 days. Samples were taken every 24 h throughout the process and a set of analysis was used to assess the performance of the enzymatic hydrolysis.

**Figure 2:**
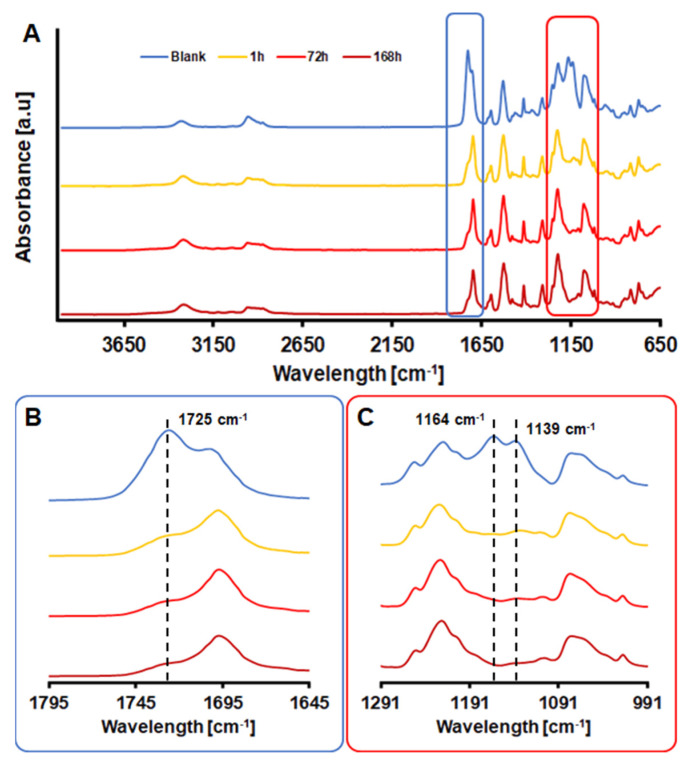
(**A**) FT-IR spectra of PU-PE films at different time points during hydrolysis by HiC (all the spectra were normalized at 1413 cm^−1^); (**B**) Particulars of the significant reduction in the peak at 1725 cm^−1^; (**C**) Particulars of the significant reduction in the peaks at 1164 cm^−1^ and 1139 cm^−1^.

To evaluate the effect of the hydrolysis on the films, FT-IR spectra at various timepoints were recorded (Figure 2A). Three peaks were of particular interest: the peak at 1725 cm^−1^ (Figure 2B), which can be assigned to the C=O stretching in aliphatic and aromatic ester groups, the peak at 1164 cm^−1^ (Figure 2C), corresponding to the C–O stretching of esters and the peak at 1139 cm^−1^ (Figure 2C), attributed to the C–O–C stretching of aliphatic and aromatic esters. All three peaks show important reductions in the signal intensity since the very beginning of the incubations, suggesting that HiC is particularly active in hydrolysing the ester moieties of the copolymer.

**Figure 3:**
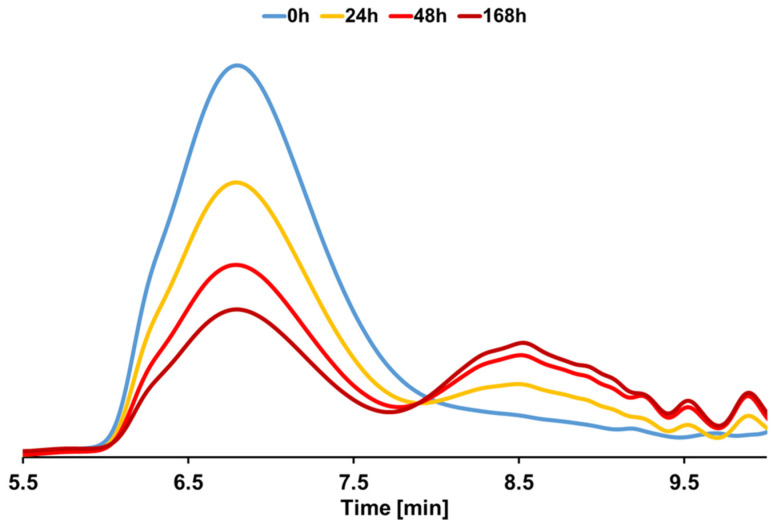
Gel permeation chromatography analysis of PU-PE films at different reaction time points during enzymatic hydrolysis by HiC.

To obtain further information on the hydrolysis of the PU-PE polymer, GPC has been performed on the residual solid part of the films after the incubation. A significant and progressive reduction in the molecular weights is shown in Figure 3, where it can be observed that, with ongoing incubation, the relative amount of molecules with relatively higher molecular weights decreases, while molecules with relatively lower molecular weights appear to be leading to a change in the signal distribution from unimodal to bimodal, thus suggesting that hydrolysis is effectively taking place. Specifically, the number average molecular weight (M_n_) decreased from 22 kDa to 3.4 kDa after 72 h with no further change after 168 h, while the weight average molecular weight (M_w_) decreased from 108 kDa to 63 kDa after 72 h, not changing significantly further after 168 h. Hence, M_n_ and M_w_ are reduced by 84% and 42%, respectively. This also causes the dispersity index (Ð) to increase from 4.89 to 17.89 after 168 h, a value more than 3 times higher. This increase is a further indication of the occurrence of an effective hydrolysis which yields highly dispersed oligomers. An extensive set of data for each specific time point can be found in Table S1 in the ESI, where the original chromatograms for each timepoint are also reported as Figures S7–S10.

**Figure 4:**
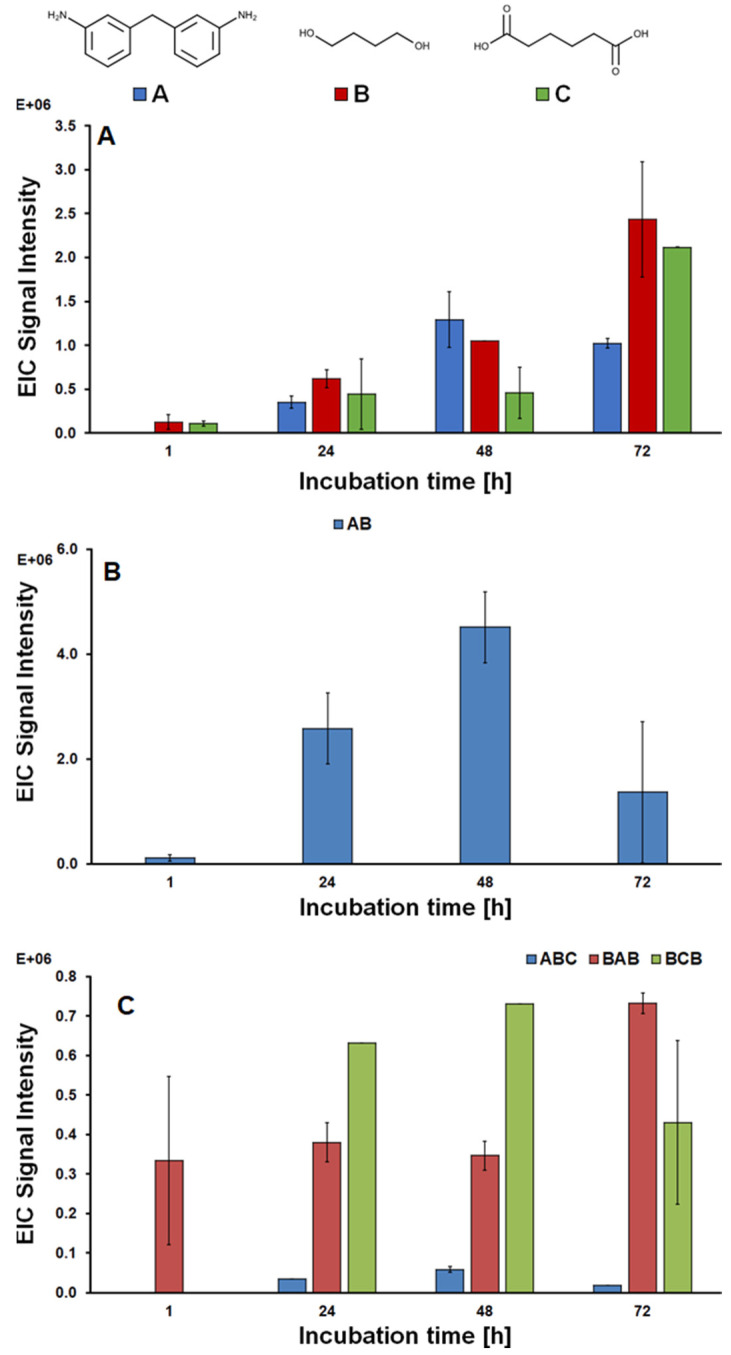
LC-TOF/MS analysis of the monomers and oligomers released in solution after the enzymatic hydrolysis of PU-PE. (**A**) monomers; (**B**) dimer; (**C**) trimers. All reactions were carried out in triplicate and presented ± the standard deviation.

Three different monomers were identified, namely 3,3′-methylendianiline (MDA), 1,4-butanediol and adipic acid. Their concentration increased throughout incubation, confirming that an effective hydrolysis took place (Figure 4A). In particular, the presence of MDA indicates that the enzyme was able to cleave not only the ester bond, but also—to a lesser extent—the urethane portion of the polymer. This observation correlates well with the distribution of the polymer observed via GPC (Figure 3) and was somehow expected since in nature, cutinases are hydrolytic enzymes whose specific function is to degrade cutin, a polymer whose monomers are linked by ester bonds. Additionally, the mechanistic aspects can be inferred observing the activity data for HiC obtained with different model substrates, pNPB and pNPA in particular (Figure 1). The release of the chromophore from pNPB takes place upon hydrolysis of an ester bond, while the chromophore group is released from pNPA after hydrolysis of an amide bond. The great difference between the activities of the enzyme obtained with the two substrates allows us to hypothesise that the enzyme preferentially hydrolyses the ester portion of the urethane bond, yielding an unstable intermediate that, upon releasing a molecule of CO_2_, is converted into MDA.

As for the dimers’ signal (Figure 4B), an increase in the first days followed by a reduction in the later stages of the hydrolysis was observed. This reduction is probably due to the fact that dimers were initially produced as products of oligomer hydrolysis and were then progressively hydrolysed, yielding monomers. The same hypothesis can be made for the trimers (Figure 4C), where the signal remained constant throughout the incubation, probably because of a combined effect of production and degradation.

**Figure 5:**
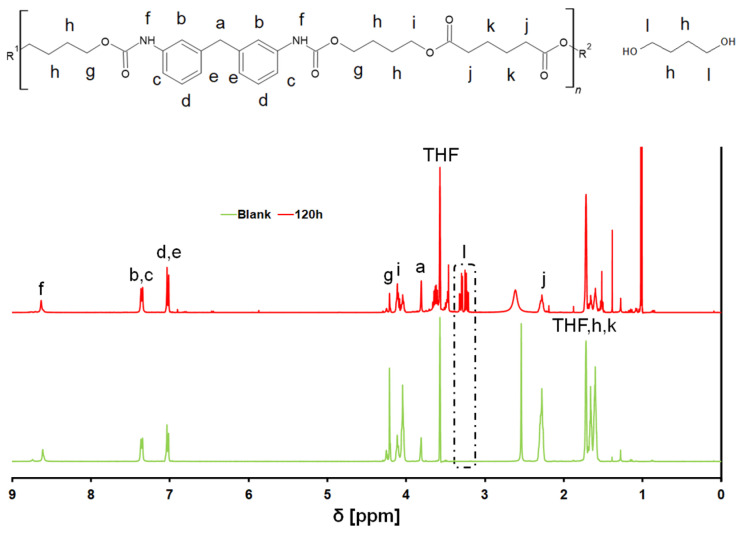
^1^H-NMR spectra of the PU-PE films before and after 120 h of incubation.

^1^H-NMR analyses were performed to identify the degradation products. An extensive analysis of the NMR spectra of diisocyanate-based polyurethanes had been performed by [42] and was very useful as a starting point for this study. Compared to the untreated polymer, the sample after 120 h of incubation showed a number of additional signals, in particular one at 3.6 ppm, assigned to the protons linked to the external carbon atoms of the free diol molecules [43], whose appearance during the incubation further confirms the hydrolysis of the ester bonds by the enzyme (Figure 5). All the signals were integrated using the integral from the methylene group of the methylendianiline as a reference, since it was sure not to react or be modified by the enzymatic treatment. Interestingly, throughout the incubation, the integrated values of the peaks at 2.3 ppm, 4.05 ppm and 4.15 ppm, assigned respectively to the protons linked to the carbons “j”, “i” and “g”, were heavily reduced. The fact that those three carbon atoms are those linked to the ester bonds further suggests that the hydrolysis took place preferentially at the ester bonds. The integrated values from the various ^1^H-NMR spectra are reported in Table S2 in the ESI.

**Figure 6:**
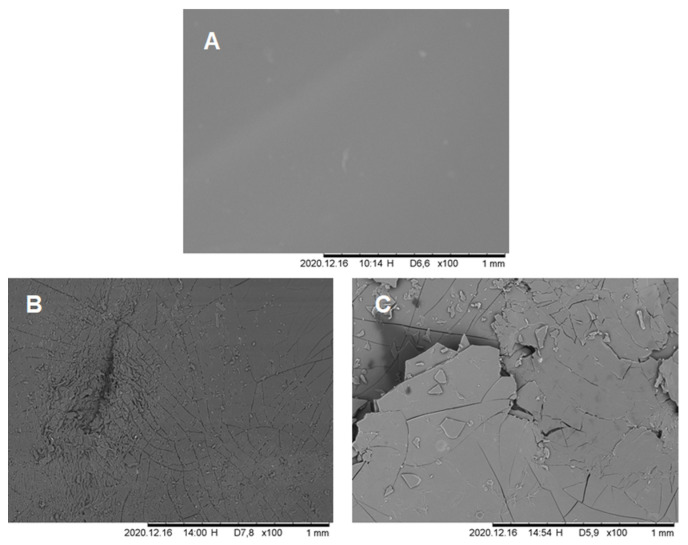
SEM images of the PU-PE films during enzymatic hydrolysis. (**A**) Blank; (**B**) 72 h of incubation; (**C**) 168 h of incubation. Magnification 100x.

A visual proof of PU-PE hydrolysis was obtained via SEM imaging on PU-PE films before (Figure 6A) and after incubation. After 72 h, deep, ramified ruptures appeared on the polymer, producing irregularly shaped fragments detaching from its surface (Figure 6B). At the end of the incubation, after 168 h, much bigger fragments can be observed, and their detachment from the film surface is much more pronounced (Figure 6C), showing a well-detectable surface erosion, as also presented in Figure S11.

## 4. Conclusions

In this study, the hydrolysis of PU-PE films by a cutinase from *Humicola insolens* was demonstrated. FT-IR analysis showed hydrolysis primarily of the ester bonds resulting in a decrease in the M_n_ and the M_w_ by 84% and 42%, respectively, as shown by the GPC analysis. Hydrolysis was also reflected by SEM, indicating significant decomposition already after 72 h of incubation. Furthermore, the presence of free diol molecules and 3,3′-methylendianiline among the degradation products, verified by ^1^H-NMR and LC-TOF/MS analysis, indicates that the substrate specificity of HiC is broad enough to make it capable of hydrolysing both the ester and the urethane bond, showing its versatility and thus making it a promising tool for the still-challenging enzymatic degradation of polyurethanes.

## Data Availability

All data are contained in the ESI or available upon email request from the authors.

## Supplementary Materials

The following are available online at https://www.mdpi.com/article/10.3390/polym14030411/s1, Figure S1: Model substrates used for the hydrolytic activity assay; (A) para-nitrophenyl-butyrate (p-NPB); (B) para-nitrophenyl-N-benzylcarbamate (p-NPC); (C) butyl-N(para-nitrophenyl)carbamate (p-NPBC); (D) para-nitrobutyranilide (p-NPA), Figure S2: LC-TOF/MS analysis of the released product 3,3’-methylenedianiline, chemical formula: C_13_H_14_N_2_. Exact mass: 198.12 Da., Figure S3: LC-TOF/MS analysis of the released product 4-hydroxybutyl (3-(3-aminobenzyl)phenyl)carbamate, chemical formula: C_18_H_22_N_2_O_3_. Exact mass: 314.16 Da., Figure S4: LC-TOF/MS analysis of the released product adipic acid, chemical formula: C_6_H_10_O_4_. Exact mass: 146.06 Da., Figure S5: LC-TOF/MS analysis of the released product bis(4-hydroxybutyl) (methylenebis(3,1-phenylene))dicarbamate, chemical formula: C_23_H_30_N_2_O_6_. Exact mass: 430.21 Da., Figure S6: Activity of assay of HiC (blue dots) in presence of para-nitrophenyl-N-benzylcarbamate as substrate. The blank (red dot) was performed in presence of only buffer., Figure S7: GPC chromatogram of the PU-PE film after 0h of incubation., Figure S8: GPC chromatogram of the residual PU-PE film after 24 h of incubation., Figure S9: GPC chromatogram of the residual PU-PE film after 48 h of incubation., Figure S10: GPC chromatogram of the residual PU-PE film after 168 h of incubation., Figure S11: SEM images of the PU-PE films during enzymatic hydrolysis. (A) Blank; (B) 72 h of incubation; (C) 168 h of incubation. Magnification 1000×. Table S1: Values of Mn, Mw and Ð throughout enzymatic hydrolysis of PU as evaluated from the GPC analysis, Table S2: Integrated values of the peaks corresponding to the carbon atoms linked to the ester bonds at the beginning and at the end of the incubation (all the peaks where manually integrated using the signal from the methylene group of the methylendianiline as a reference).
