# Supplementary material for: Cutinase-Catalyzed Polyester-Polyurethane Degradation: Elucidation of the Hydrolysis Mechanism
Source: Polymers (Basel). 2022 Jan 20;14(3):411. doi: 10.3390/polym14030411 (PMC8838978; doi:10.3390/polym14030411)
Supplement: Supplementary file 1 [file polymers-14-00411-s001.zip › polymers-1519583-supplementary.pdf]

polymers
MDPI

Electronic Supplementary Information

Cutinase-Catalyzed Polyester-Polyurethane Degradation: Elucidation of the Hydrolysis Mechanism

Federico Di Bisceglie [1], Felice Quartinello [1,2,*], Robert Vielnascher [1], Georg M. Guebitz [1,2] and Alessandro Pellis [1,2,3,*]

[1] Department of Agrobiotechnology, University of Natural Resources and Life Sciences Vienna, 3430 Tulln an der Donau, Austria; federico.dibisceglie@gmail.com (F.D.B.); robert.vielnascher@boku.ac.at (R.V.); guebitz@boku.ac.at (G.M.G.)
[2] Austrian Centre of Industrial Biotechnology, 3430 Tulln an der Donau, Austria
[3] Dipartimento di Chimica e Chimica Industriale, Universitá degli Studi di Genova, Via Dodecaneso 31, 16146 Genova, Italy
* Correspondence: felice.quartinello@boku.ac.at (F.Q.); alessandro.pellis@boku.ac.at (A.P.)

Citation: Di Bisceglie, F.; Quartinello, F.; Vielnascher, R.; Guebitz, G.M.; Pellis, A. Cutinase-Catalyzed Polyester-Polyurethane Degradation: Elucidation of the Hydrolysis Mechanism. *Polymers* **2022**, *14*, 411. https://doi.org/10.3390/polym14030411

Academic Editor: Linbo Wu

Received: 10 December 2021
Accepted: 17 January 2022
Published: 20 January 2022

Publisher's Note: MDPI stays neutral with regard to jurisdictional claims in published maps and institutional affiliations.

Copyright: © 2022 by the authors. Licensee MDPI, Basel, Switzerland. This article is an open access article distributed under the terms and conditions of the Creative Commons Attribution (CC BY) license (http://creativecommons.org/licenses/by/4.0/).

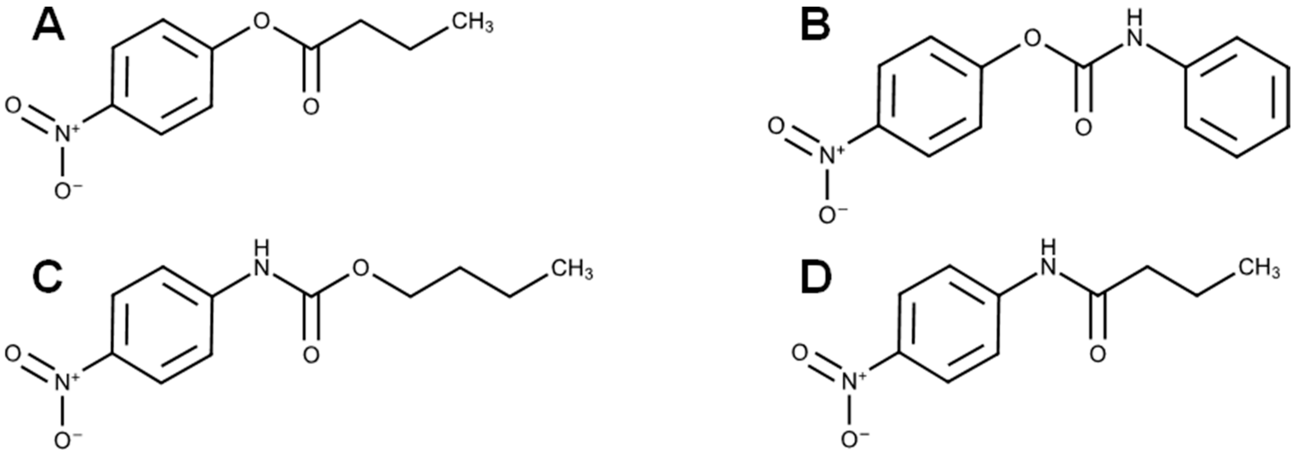

Figure S1. Model substrates used for the hydrolytic activity assay; (**A**) para-nitrophenyl-butyrate (pNPB); (**B**) para-nitrophenyl-N-benzylcarbamate (pNPC); (**C**) butyl-N(para-nitrophenyl)carbamate (pNPBC); (**D**) para-nitrobutyranilide (pNPA).

Table S1. Values of M_n, M_w and Đ before and after enzymatic hydrolysis of PU as evaluated via GPC analysis.

	M_n	M_w	Đ
**T0**	22000	107800	4.89
**24 h**	6400	90600	14.20
**48 h**	4100	74000	17.96
**72 h**	3400	62700	18.38
**120 h**	3600	64700	17.99
**168 h**	3500	62200	17.89

Table S2. Integrated values of the peaks corresponding to the carbon atoms linked to the ester bonds at the beginning and at the end of the incubation (all the peaks where manually integrated using the signal from the methylene group of the methylendianiline as the reference).

**δ (ppm)**	2.3 (“j”)	4.05 (“i”)	4.15 (“g”)
**Blank**	13.6429	12.4187	5.3357
**T0**	11.5995	10.8860	4.6157
**24 h**	7.7351	8.4851	3.0704
**48 h**	5.9597	7.3946	2.4300
**72 h**	4.7645	6.4077	1.9587
**120 h**	3.2922	5.5477	1.2741
**168 h**	5.2897	7.0965	2.1912

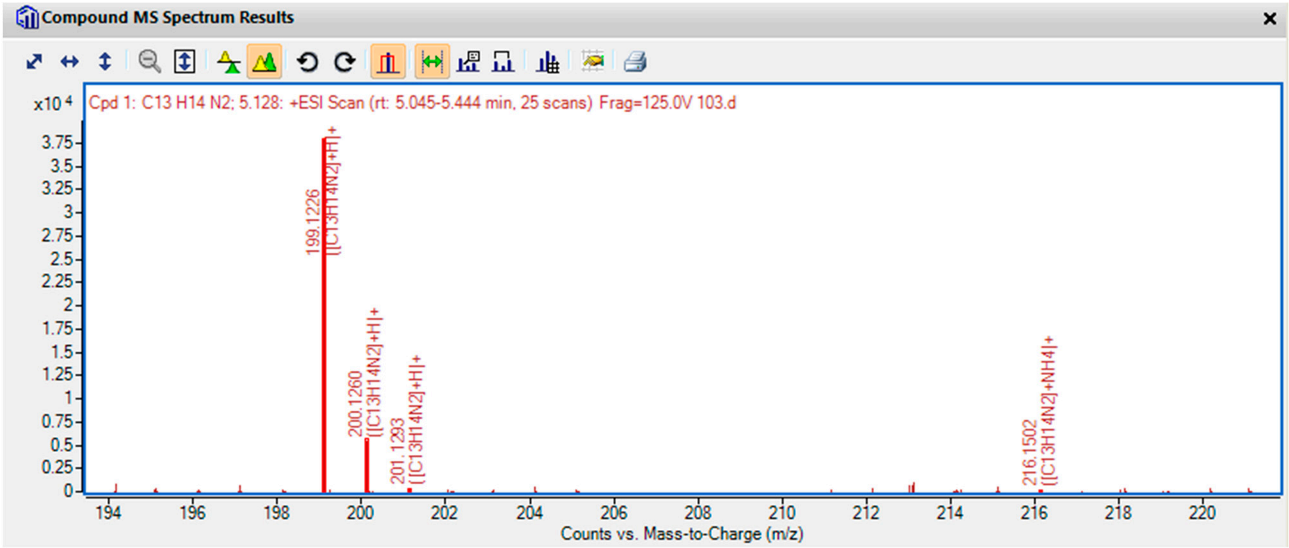

Figure S2. LC-TOF/MS analysis of the released product 3,3'-methylenedianiline, chemical formula: $C_{13}H_{14}N_2$. Exact mass: 198.12 Da.

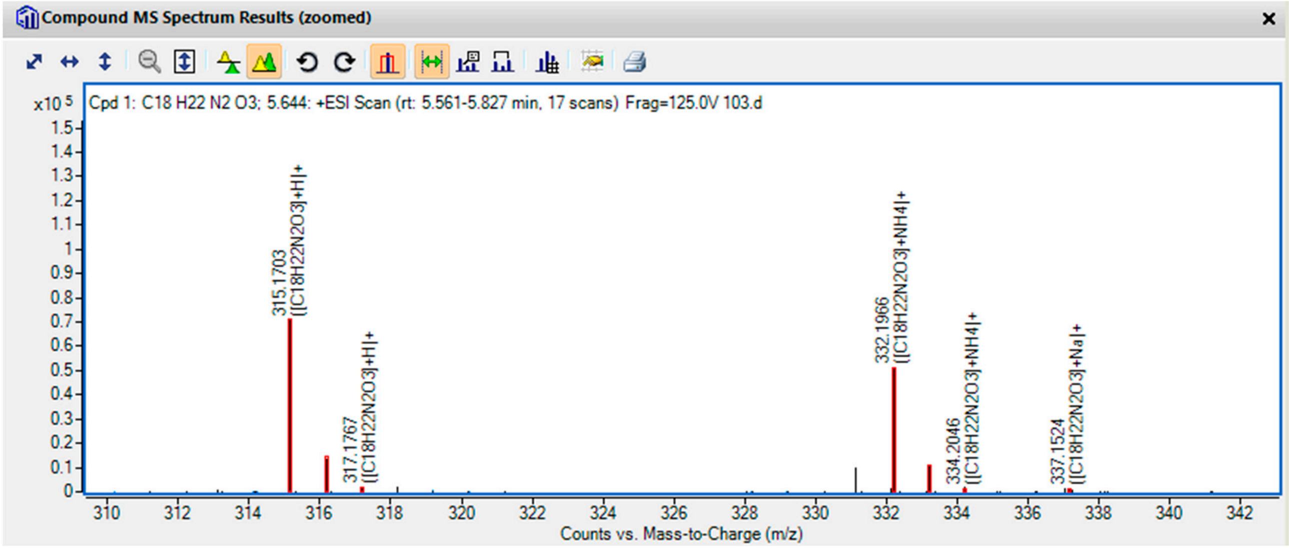

Figure S3. LC-TOF/MS analysis of the released product 4-hydroxybutyl (3-(3-aminobenzyl)phenyl)carbamate, chemical formula: $C_{18}H_{22}N_2O_3$. Exact mass: 314.16 Da.

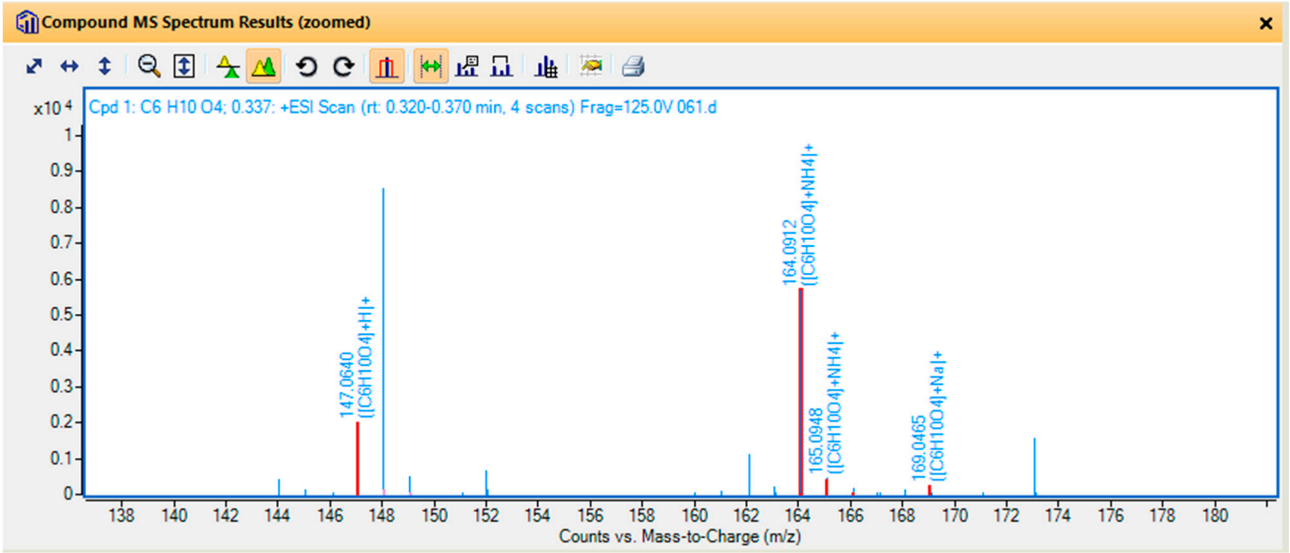

Figure S4. LC-TOF/MS analysis of the released product adipic acid, chemical formula: $C_6H_{10}O_4$. Exact mass: 146.06 Da.

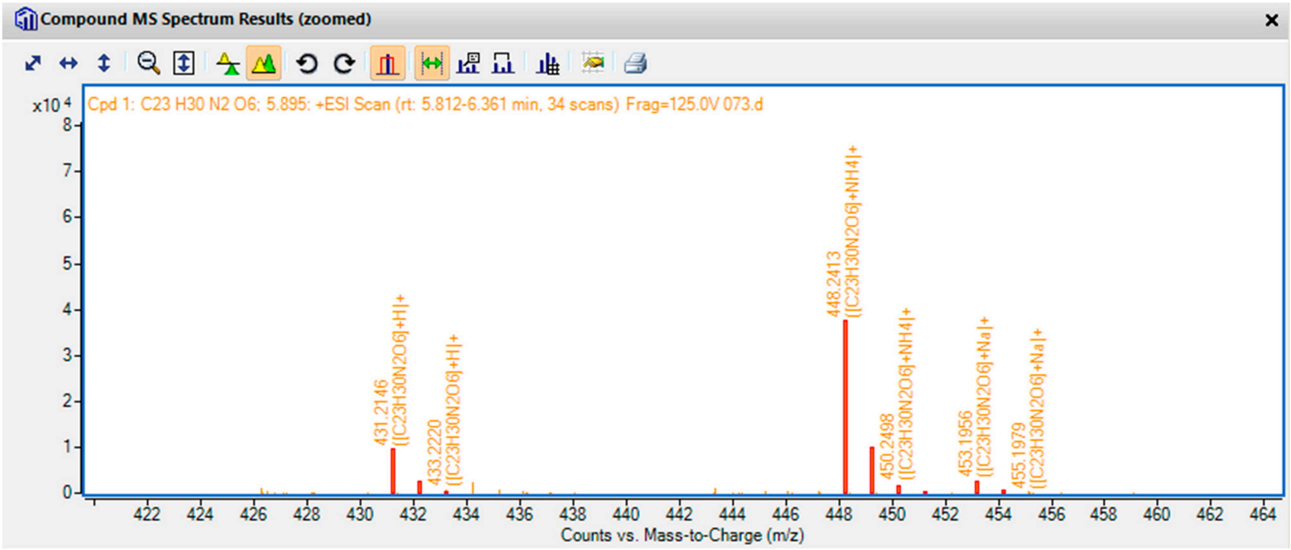

Figure S5. LC-TOF/MS analysis of the released product bis(4-hydroxybutyl) (methylenebis(3,1-phenylene))dicarbamate, chemical formula: $C_{23}H_{30}N_2O_6$. Exact mass: 430.21 Da.

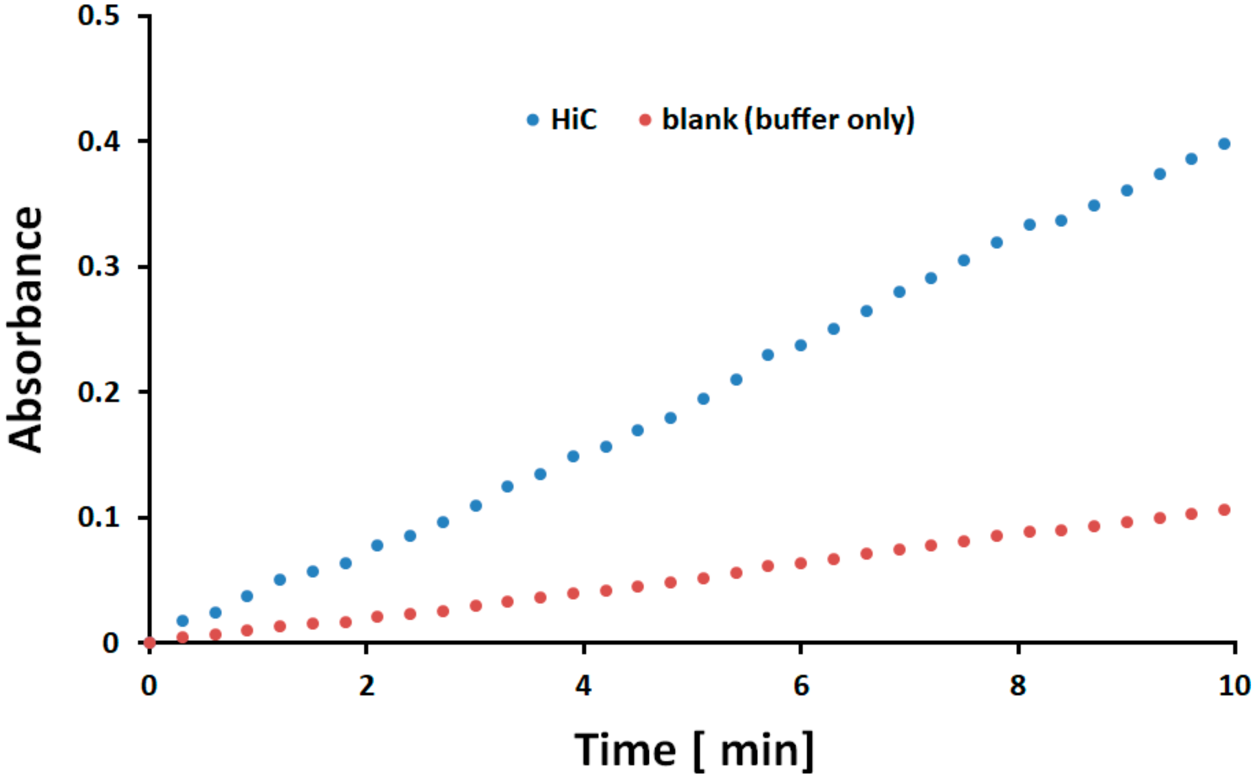

Figure S6. Activity of assay of HiC (blue dots) in presence of para-nitrophenyl-N-benzylcarbamate as substrate. The blank (red dot) was performed in presence of only buffer.

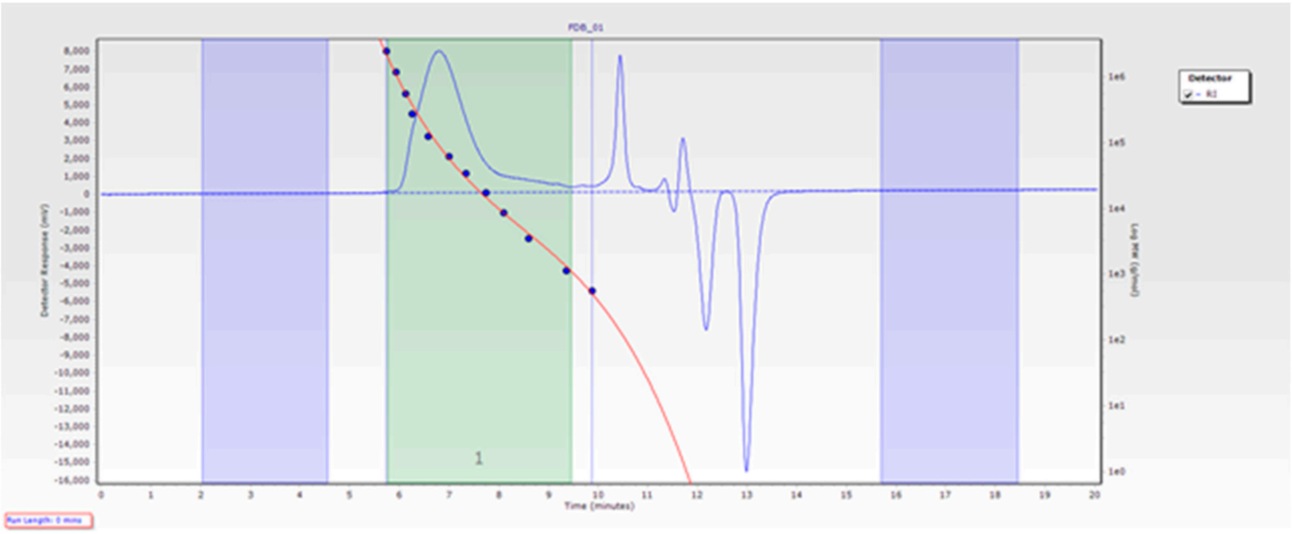

Figure S7. GPC chromatogram of the PU-PE film after 0h of incubation

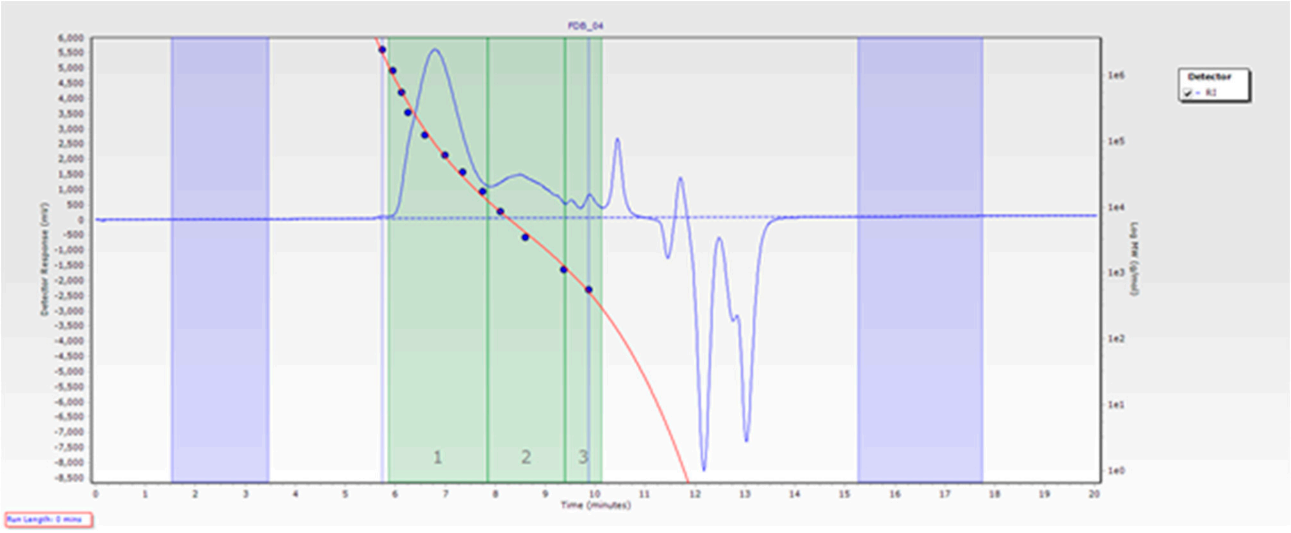

Figure S8. GPC chromatogram of the residual PU-PE film after 24h of incubation

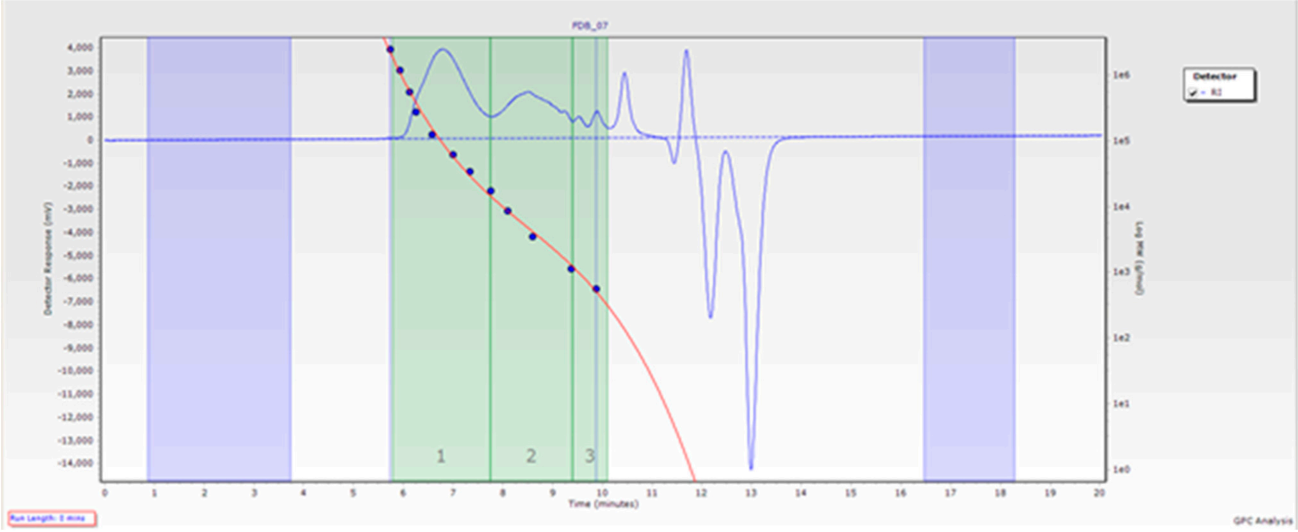

Figure S9. GPC chromatogram of the residual PU-PE film after 48h of incubation

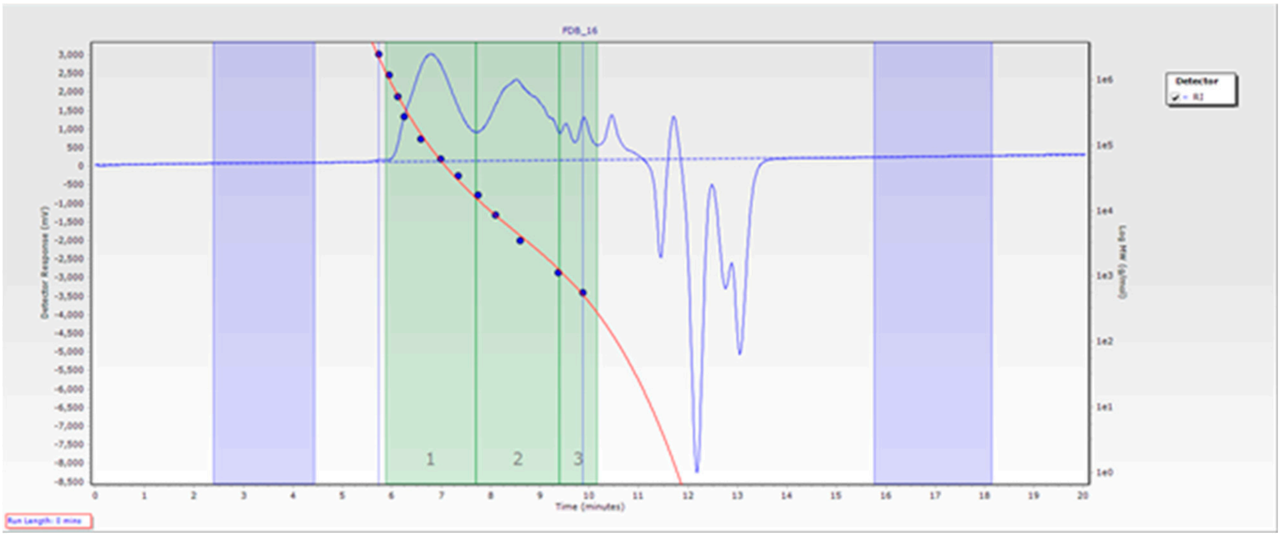

Figure S10. GPC chromatogram of the residual PU-PE film after 168h of incubation

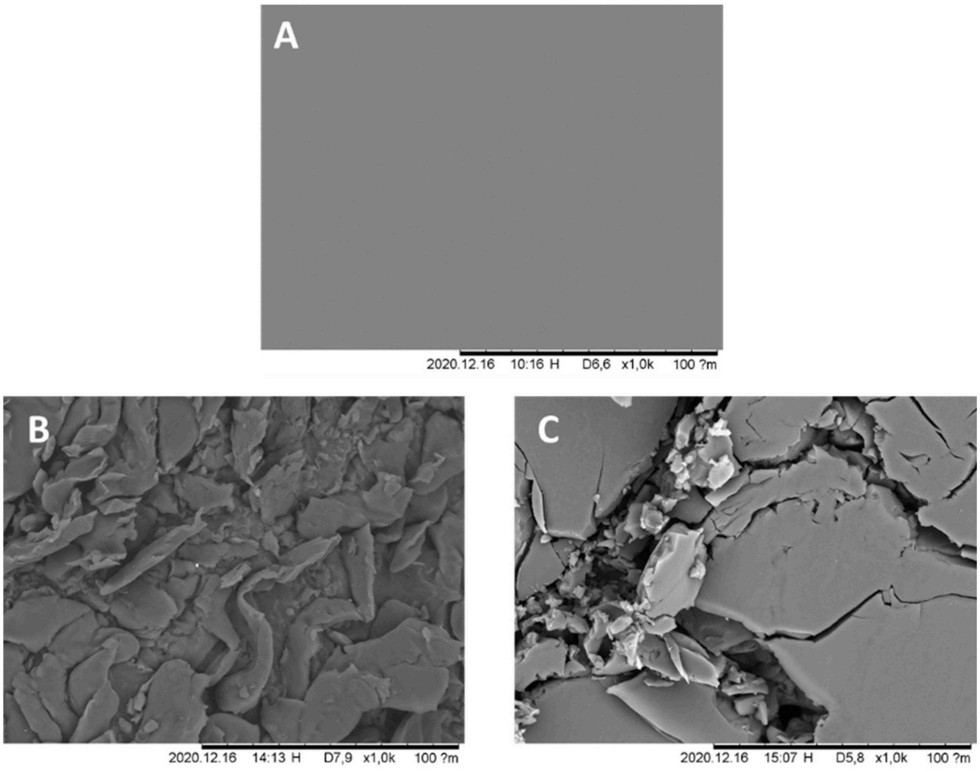

Figure S11. SEM images of the PU-PE films during enzymatic hydrolysis. (**A**) Blank; (**B**) 72 h of incubation; (**C**) 168 h of incubation. Magnification 1000x.
